# New Mexico’s aggressive plan to end child food insecurity and hunger through universal school meals policy adoption

**DOI:** 10.1017/S1368980024002726

**Published:** 2025-01-09

**Authors:** Olivia M Thompson

**Affiliations:** The University of New Mexico, School of Medicine, College of Population Health, 1155 University Blvd. S, Albuquerque, NM 87131, USA

**Keywords:** Universal school meals, Food insecurity, Hunger, Nutrition policy, Childhood obesity

States throughout the USA have begun preparing for the roll-out of new and improved United States Department of Agriculture (USDA) school nutrition requirements that limit the amount of added sugars and Na allowed in foods and beverages and increase menu planning flexibilities, particularly for traditional Indigenous foods^(1)^. Beginning in the 2025/2026 school year, the amount of added sugars allowed in breakfast cereals, yogurt and flavoured milk will be reduced; and beginning in the 2027/2028 school year, the amount of added sugars allowed across the school week will be limited to less than 10 % of total calories. Also beginning in the 2025/2026 school year, schools will start gradually reducing the amount of Na in both lunch and breakfast meals, by a total of 15 % in lunch meals and 10 % in breakfast meals no later than the beginning of the 2027/2028 school year.

Currently, schools are able to offer an increased variety of appealing foods for breakfasts, lunches, dinners and afterschool snacks. For example, schools are able to offer grains, meat or meat alternates (i.e. beans, peas, lentils, cheese, eggs, nuts, seeds, tempeh, tofu and yogurt), or a combination of both grains and meat or meat alternates to meet nutritional requirements for school breakfasts. Additionally, for breakfasts, schools are able to substitute vegetables for fruits. Now schools can substitute any vegetables for fruits regardless of how many times per week such substitution is made; however, beginning in the 2025/2026 school year, if vegetables are substituted for fruits 1 d per week, schools will be able to offer any type of vegetable and if vegetables are substituted for fruits two or more days per week, schools will be able to offer vegetables from one of four subgroups (i.e. dark green; red/orange; beans, peas, and lentils; and other vegetables).

Schools that are tribally operated, operated by the Bureau of Indian Education, and that serve primarily American Indian or Alaska Native children can substitute vegetables for grains at any meal. Any type of school (tribally controlled or otherwise) can incorporate traditional Indigenous foods into their school meal programmes and serve foods in reimbursable school meals. Traditional Indigenous foods are defined by the USDA as ‘food that has traditionally been prepared and consumed by an Indian tribe’ and include wild game meat, fish, seafood, marine animals, plants and berries. Popular, kid-tested recipes such as Pinto Bean Dip with Roasted Pine Nuts, Corn Chowder with Wild Plantains and Salmon, and Bison Meatballs with Dandelion Tomato Sauce are available on the USDA’s website^(2)^.

New Mexico is particularly equipped to not only implement the new and improved USDA school nutrition requirements, but New Mexico will be able to go above and beyond. On 1 July 2023, in an effort to dampen New Mexico’s child food insecurity and hunger exigency, Gov. Michelle Lujan Grisham signed Senate Bill 4 (SB 4), which established the Healthy Hunger Free Students Bill of Rights Act^(3)^. This Act, a now $41 million dollar investment^(4)^, ensures that kindergarten through 12th grade (k-12) students enrolled in operating public and public charter schools (including tribally controlled and private schools that opt in), regardless of their household income, have free-of-cost meals beginning in the 2023/2024 school year, with meal quality standards and food waste guidelines being phased in over 2 years. Meal quality standards include (1) purchasing New Mexico-produced foods or food products, (2) freshly preparing meals using scratch-cooking methods, (3) providing culturally relevant meals and (4) engaging students and their families in choices for menu development. Food waste guidelines include (1) students in grades k-5 shall be allowed to have up to 20 min of seated lunch and (2) share tables shall be provided whereby allowable food shall be donated to students, food banks or other non-profit charitable organisations each school day.

Including New Mexico, universal free school meal policies are now permanently adopted in eight states (i.e. California, Maine, Colorado, Minnesota, New Mexico, Vermont, Massachusetts and Michigan), and studies highlighting policy impacts on child education and health outcomes are extremely positive^(5,6)^. Given these positive impacts, New Mexico has, just this past September, strengthened provisions outlined in Senate Bill (SB) 4 by adopting a rule that establishes the standards and procedures for SB 4 implementation required for schools to receive an official certification to establish a ‘Healthy Universal School Meals Program’, on or before 1 July 2025^(7)^. (During the 2024/2025 school year, 77 % or a total of 902 schools (out of approximately 1173 eligible public, public charter, tribally controlled and private schools) applied to participate in the Healthy Universal School Meals Program^(4)^). Importantly, standards include meal quality improvements for meals to be prepared and cooked for same day consumption using ‘scratch’ or ‘speed scratch’ preparation methods. In other words, meals will be prepared using whole, fresh ingredients that include raw proteins, whole grains, and fresh fruits and vegetables or made by blending fresh ingredients together with pre-prepared and ready-made, minimally processed ingredients. New Mexico schools will also be eligible to receive incentive grants to purchase New Mexican-grown, raised or processed foods and food products, with a minimum of 75 % of funds used to purchase unprocessed and minimally processed products, and with up to 25 % of funds used to purchase value-added processed products. By 1 August of each year, certified schools will receive the greater of one thousand dollars or an amount equal to 10 cents multiplied by the number of lunches qualified for federal free meal reimbursement served to students during the preceding school year. Time will tell if such monetary incentive will be enough to offset the costs of SB 4 implementation and enable school food service operations to fully implement policy provisions and thus realise New Mexico’s significant financial investment in ensuring that all children, regardless of their ability to pay, have access to nutritious meals while at school.
